# Supplementary material for: Ecosystem-wide metagenomic binning enables prediction of ecological niches from genomes
Source: Commun Biol. 2020 Mar 13;3:119. doi: 10.1038/s42003-020-0856-x (PMC7070063; doi:10.1038/s42003-020-0856-x)
Supplement: Supplementary file 1 — Supplementary Information [file 42003_2020_856_MOESM1_ESM.pdf]

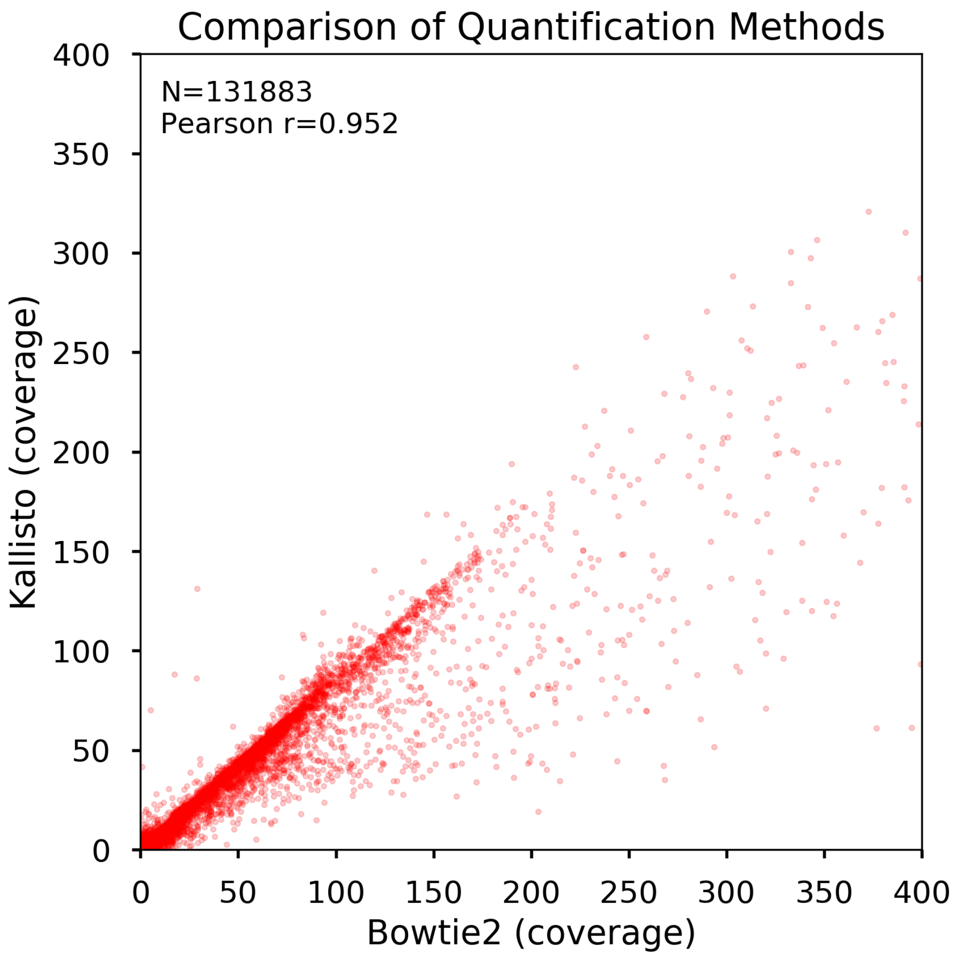

Supplementary Figure 1. Comparison of contig quantifications obtained with Kallisto and Bowtie2. Each point corresponds to one contig; all contigs derive from the same metagenome sample. The reads that were used for quantification come from another sample.

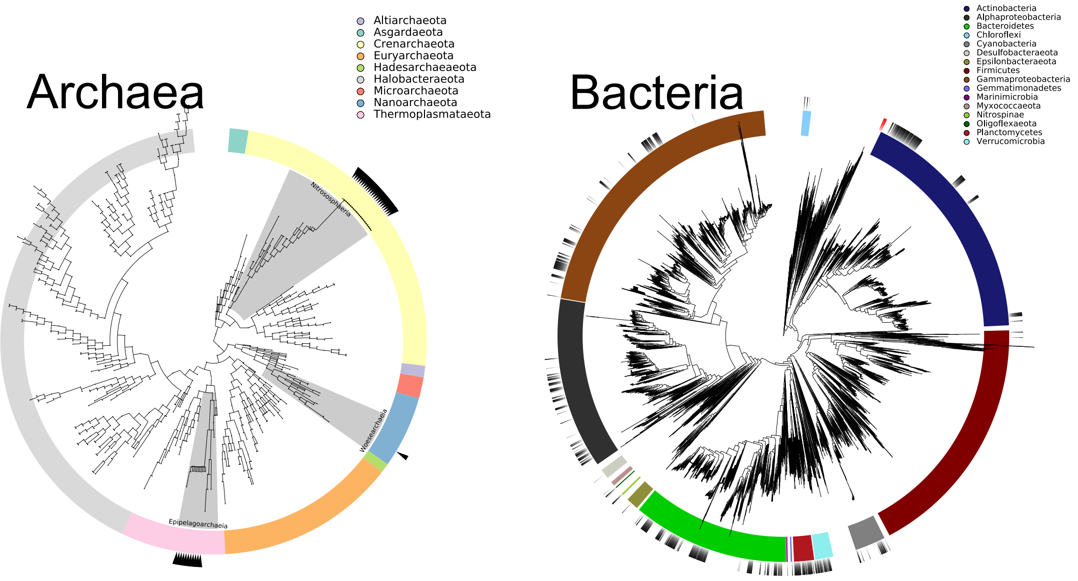

Supplementary Figure 2. Genome Taxonomy Database (GTDB) phylogenetic trees with the MAGs obtained in this study inserted. *Thermus thermophilus* MAGs obtained from the spike-in DNA are indicated with red arrows, all other MAGs with black arrows.

			CheckM estimates (in percentage)			Mummer alignments against reference genome (in percentage)					
						Chromosome		Plasmid pTT27		Plasmid pTT8	
ID	MAG length (bp)	n50_len (bp)	Completeness	Contam.	Strain heterogen.	Aligned ref. bases	Average identity	Aligned ref. bases	Average identity	Aligned ref. bases	Average identity
P1994_101_bin107	2169312	92640	99.58	0	0	99.83	99.98	99.51	99.96	100	100
P1994_102_bin142	2165903	91962	99.58	0	0	99.53	99.97	99.42	99.96	100	100
P1994_103_bin65	2177942	94990	99.58	0	0	99.49	99.98	99.48	99.83	100	100
P1994_104_bin21	2170841	94897	99.58	0	0	99.99	99.97	99.64	99.97	100	100
P1994_105_bin111	1864526	16199	86.05	0	0	86.18	99.94	88.95	99.96	10.13	99.26
P1994_106_bin14	2173387	77393	99.58	0	0	99.93	99.98	99.44	99.97	100	100
P1994_107_bin50	1858350	3851	84.6	0.94	25	85.63	99.93	89.44	99.78	100	100
P1994_108_bin218	2165889	94911	99.58	0	0	99.48	99.97	99.54	99.77	100	100
P1994_110_bin108	2170433	94895	99.58	0	0	99.77	99.97	99.55	99.76	100	100
P1994_111_bin90	2166422	91036	99.58	0	0	99.95	99.96	99.66	99.96	100	100
P1994_112_bin6	2170845	91876	99.58	0	0	99.99	99.96	99.61	99.95	100	100
P1994_113_bin206	2169068	84441	98.94	0	0	99.98	99.97	99.26	99.91	100	100
P1994_114_bin162	2169614	68004	99.58	0	0	99.94	99.98	99.76	99.78	100	100
P1994_115_bin22	2159853	19991	98.52	0.42	100	99.78	99.94	98.46	99.94	100	100
P1994_116_bin172	2167853	94918	99.58	0	0	99.30	99.98	98.42	99.84	100	100
P1994_117_bin132	2169952	94918	99.58	0	0	99.94	99.98	99.14	99.94	100	100
P1994_118_bin6	2168350	92060	99.58	0	0	99.76	99.98	98.27	99.98	100	100
P1994_119_bin191	2169969	91977	99.36	0	0	99.96	99.96	99.61	99.83	100	100
P1994_120_bin89	2168245	94919	99.58	0	0	99.81	99.98	99.30	99.81	100	100
P1994_121_bin88	2165532	119112	99.58	0	0	99.73	99.97	99.17	99.99	100	99.92
P1994_122_bin164	2170746	92064	99.58	0	0	99.81	99.98	99.42	99.99	100	100
P1994_123_bin13	2166444	94918	99.58	0	0	99.81	99.98	98.00	99.71	100	99.97
P1994_124_bin173	2162444	94919	99.58	0	0	99.37	99.98	98.96	99.99	100	100
P1994_125_bin183	2170378	91955	99.58	0	0	99.94	99.97	99.59	99.98	100	100
P1994_126_bin183	2171116	94919	99.58	0	0	99.85	99.97	98.55	99.95	100	100
P1994_127_bin47	2159860	52869	99.36	0	0	99.52	99.95	98.82	99.86	100	100
P1994_129_bin128	2169229	94919	99.58	0	0	99.83	99.98	98.58	99.94	100	100
P1994_130_bin6	2170618	94919	99.58	0	0	99.94	99.97	99.49	99.83	100	100

**Reference segment**	**Length (bp)**
Chromosome	1849742
Plasmid pTT27	256992
Plasmid pTT8	9322
Total	2116056

Supplementary Table 1. Assembly statistics, CheckM genome quality estimates, and statistics from aligning the 28 different *T. thermophilus* MAGs to the reference genome (*T. thermophilus* HB8). The different segments were aligned to separately using MUMmer.

		Prediction method			
**Variable**	**Variance explained**	**Phylogenetic**	**Ridge regression**	**Random forests**	**Gradient boosting**
PC1	0.11	0.48	0.72	0.71	0.74
PC2	0.05	0.68	0.68	0.70	0.70
PC3	0.04	0.60	0.68	0.65	0.64
PC4	0.02	0.57	0.67	0.67	0.66
PC5	0.02	0.65	0.75	0.73	0.79
PC6	0.02	0.49	0.60	0.61	0.64
PC7	0.01	0.69	0.68	0.70	0.69
PC8	0.01	0.51	0.51	0.47	0.54
PC9	0.01	0.40	0.38	0.44	0.46
PC10	0.01	0.64	0.57	0.61	0.60
high sal-to-low sal ratio		0.67	0.74	0.72	0.70
surface-to-mid ratio		0.62	0.76	0.77	0.81
3um-to-0.8um ratio		0.76	0.73	0.73	0.75

Supplementary Table 2. Results from ecological niche predictions. The first ten variables are the scores for first ten principal coordinates from the PCoA on the BACL abundance profiles, with proportion of variation explained by the principal coordinates indicated. The last three variables are the niche variables illustrated in Fig. 3 (log ratio between abundance in high and low salinity samples, log ratio between abundance in surface and mid layer, and log ratio between abundance on 3.0 µm and 0.8 µm filters). For each prediction method the Spearman correlation coefficient is given between the predicted and observed value.

	#BACL	Variance explained	Prediction method			
			**Phylogenetic**	**Ridge regression**	**Random forests**	**Gradient boosting**
**PC1**						
p__Actinobacteria	68	0.22	0.40	0.55	0.70	0.67
p__Bacteroidetes	83	0.23	0.32	0.73	0.75	0.78
c__Alphaproteobacteria	43	0.24	0.22	0.35	0.46	0.33
c__Gammaproteobacteria	54	0.25	0.44	0.72	0.67	0.71
**PC2**						
p__Actinobacteria	68	0.09	0.52	0.62	0.60	0.62
p__Bacteroidetes	83	0.08	0.33	0.48	0.58	0.60
c__Alphaproteobacteria	43	0.14	0.62	0.63	0.58	0.53
c__Gammaproteobacteria	54	0.10	0.69	0.52	0.59	0.67
**PC3**						
p__Actinobacteria	68	0.06	0.50	0.58	0.49	0.54
p__Bacteroidetes	83	0.05	0.42	0.61	0.52	0.53
c__Alphaproteobacteria	43	0.10	0.64	0.58	0.69	0.77
c__Gammaproteobacteria	54	0.08	0.51	0.32	0.45	0.53

Supplementary Table 3. Results from predicting the first three principal coordinate scores from PCoAs on BACL abundance profiles run on one taxonomic group at a time, with number of BACLs included and proportion of variation explained by the principal coordinates indicated. Spearman correlation coefficients between predicted and observed values are given for each prediction method. "p__" stands for phylum and "c__" class in the GTDB taxonomy.
