# Supplementary material for: Ecosystem-wide metagenomic binning enables prediction of ecological niches from genomes
Source: Commun Biol. 2020 Mar 13;3:119. doi: 10.1038/s42003-020-0856-x (PMC7070063; doi:10.1038/s42003-020-0856-x)
Supplement: Supplementary file 3 — Description of Additional Supplementary Files [file 42003_2020_856_MOESM3_ESM.pdf]

Description of Additional Supplementary Files

File Name: **Supplementary Data 1**
Description: Taxonomic classification and summary statistics for the MAGs
