# Supplementary material for: Ecosystem-wide metagenomic binning enables prediction of ecological niches from genomes
Source: Commun Biol. 2020 Mar 13;3:119. doi: 10.1038/s42003-020-0856-x (PMC7070063; doi:10.1038/s42003-020-0856-x)
Supplement: Supplementary file 5 — Reporting Summary [file 42003_2020_856_MOESM5_ESM.pdf]

nature research

Corresponding author(s): Anders Andersson

Last updated by author(s): Jul 31, 1974

Reporting Summary

Nature Research wishes to improve the reproducibility of the work that we publish. This form provides structure for consistency and transparency in reporting. For further information on Nature Research policies, see Authors & Referees and the Editorial Policy Checklist.

Statistics

For all statistical analyses, confirm that the following items are present in the figure legend, table legend, main text, or Methods section.

n/a	Confirmed	
☐	☒	The exact sample size (*n*) for each experimental group/condition, given as a discrete number and unit of measurement
☐	☒	A statement on whether measurements were taken from distinct samples or whether the same sample was measured repeatedly
☐	☒	The statistical test(s) used AND whether they are one- or two-sided<br>*Only common tests should be described solely by name; describe more complex techniques in the Methods section.*
☒	☐	A description of all covariates tested
☐	☒	A description of any assumptions or corrections, such as tests of normality and adjustment for multiple comparisons
☐	☒	A full description of the statistical parameters including central tendency (e.g. means) or other basic estimates (e.g. regression coefficient) AND variation (e.g. standard deviation) or associated estimates of uncertainty (e.g. confidence intervals)
☐	☒	For null hypothesis testing, the test statistic (e.g. *F*, *t*, *r*) with confidence intervals, effect sizes, degrees of freedom and *P* value noted<br>*Give P values as exact values whenever suitable.*
☒	☐	For Bayesian analysis, information on the choice of priors and Markov chain Monte Carlo settings
☒	☐	For hierarchical and complex designs, identification of the appropriate level for tests and full reporting of outcomes
☐	☒	Estimates of effect sizes (e.g. Cohen's *d*, Pearson's *r*), indicating how they were calculated

Our web collection on statistics for biologists contains articles on many of the points above.

Software and code

Policy information about availability of computer code

Data collection	No software was used for data collection.
Data analysis	Only open source software were used. All software used are listed, with versions, in the Methods section.

For manuscripts utilizing custom algorithms or software that are central to the research but not yet described in published literature, software must be made available to editors/reviewers. We strongly encourage code deposition in a community repository (e.g. GitHub). See the Nature Research guidelines for submitting code & software for further information.

Data

Policy information about availability of data

All manuscripts must include a data availability statement. This statement should provide the following information, where applicable:

- Accession codes, unique identifiers, or web links for publicly available datasets
- A list of figures that have associated raw data
- A description of any restrictions on data availability

The contigs from the individual samples and the MAG sequences were submitted to ENA hosted by EMBL-EBI under the study accession number PRJEB34883. Note that contigs stemming from the internal standards genome (Thermus thermophilus) are included in the contigs for the Transect 2014 s[illegible]es. The preprocessed sequencing reads from the LMO Time Series 2013-2014 and Coastal Transect 2015 samples were submitted to ENA under the same stu[illegible]cession number (PRJEB34883).The preprocessed sequencing reads from the Transect 2014 and Redoxcline 2014 samples were published elsewhere31 and are available at ENA under the study accession number PRJEB22997. The raw sequencing reads from the Askö Time Series 2011 were published elsewhere58 and are available at NCBI under the study accession number SRP077551.

Field-specific reporting

Please select the one below that is the best fit for your research. If you are not sure, read the appropriate sections before making your selection.

☐ Life sciences ☐ Behavioural & social sciences ☒ Ecological, evolutionary & environmental sciences

For a reference copy of the document with all sections, see nature.com/documents/nr-reporting-summary-flat.pdf

Ecological, evolutionary & environmental sciences study design

All studies must disclose on these points even when the disclosure is negative.

Study description	Metagenomic binning was conducted on 123 water samples spanning major environmental gradients of the Baltic Sea. The resulting 1961 metagenome-assembled genomes represented 352 species-level clusters. We tested whether the placement of each genome cluster could be predicted along various a priori defined niche gradients (salinity level, depth, size fraction) based solely on its content of functional genes using machine-learning. The same approach predicted the placement of the genome clusters in a virtual niche-space that was constructed by ordination of the clusters' abundance profiles across samples. The predictions were in most cases better than those inferred based on phylogenetic information.
Research sample	123 water samples from the Baltic Sea.
Sampling strategy	The samples represent a collection of 5 sample-sets.
Data collection	Water was sampled and filtered. DNA extracted from filter.
Timing and spatial scale	Time-scale: 2011-2015. Spatial-scale: Bay of Bothnia - Skagerrak
Data exclusions	No data was excluded
Reproducibility	We included an internal-spike in genome in a subset of samples to evaluate the accuracy of the assembly and binning approach
Randomization	When conducting machine-learning, genome-clusters where randomly selected for training or testing, respectively
Blinding	Not relevant here

Did the study involve field work? ☒ Yes ☐ No

Field work, collection and transport

Field conditions	These data are provided in the manuscript.
Location	These data are provided in the manuscript.
Access and import/export	The samples were colleced and exported in compliance with national and international laws.
Disturbance	Sampling was conducted in pelagic waters without disturbing animals or humans.

Reporting for specific materials, systems and methods

We require information from authors about some types of materials, experimental systems and methods used in many studies. Here, indicate whether each material, system or method listed is relevant to your study. If you are not sure if a list item applies to your research, read the appropriate section before selecting a response.

Materials & experimental systems

n/a	Involved in the study	
☒	☐	Antibodies
☒	☐	Eukaryotic cell lines
☒	☐	Palaeontology
☒	☐	Animals and other organisms
☒	☐	Human research participants
☒	☐	Clinical data

Methods

n/a	Involved in the study	
☒	☐	ChIP-seq
☒	☐	Flow cytometry
☒	☐	MRI-based neuroimaging
